# Supplementary material for: Machilin A Inhibits Tumor Growth and Macrophage M2 Polarization Through the Reduction of Lactic Acid
Source: Cancers (Basel). 2019 Jul 9;11(7):963. doi: 10.3390/cancers11070963 (PMC6678097; doi:10.3390/cancers11070963)
Supplement: Supplementary file 1 [file cancers-11-00963-s001.pdf]

Supplementary materials

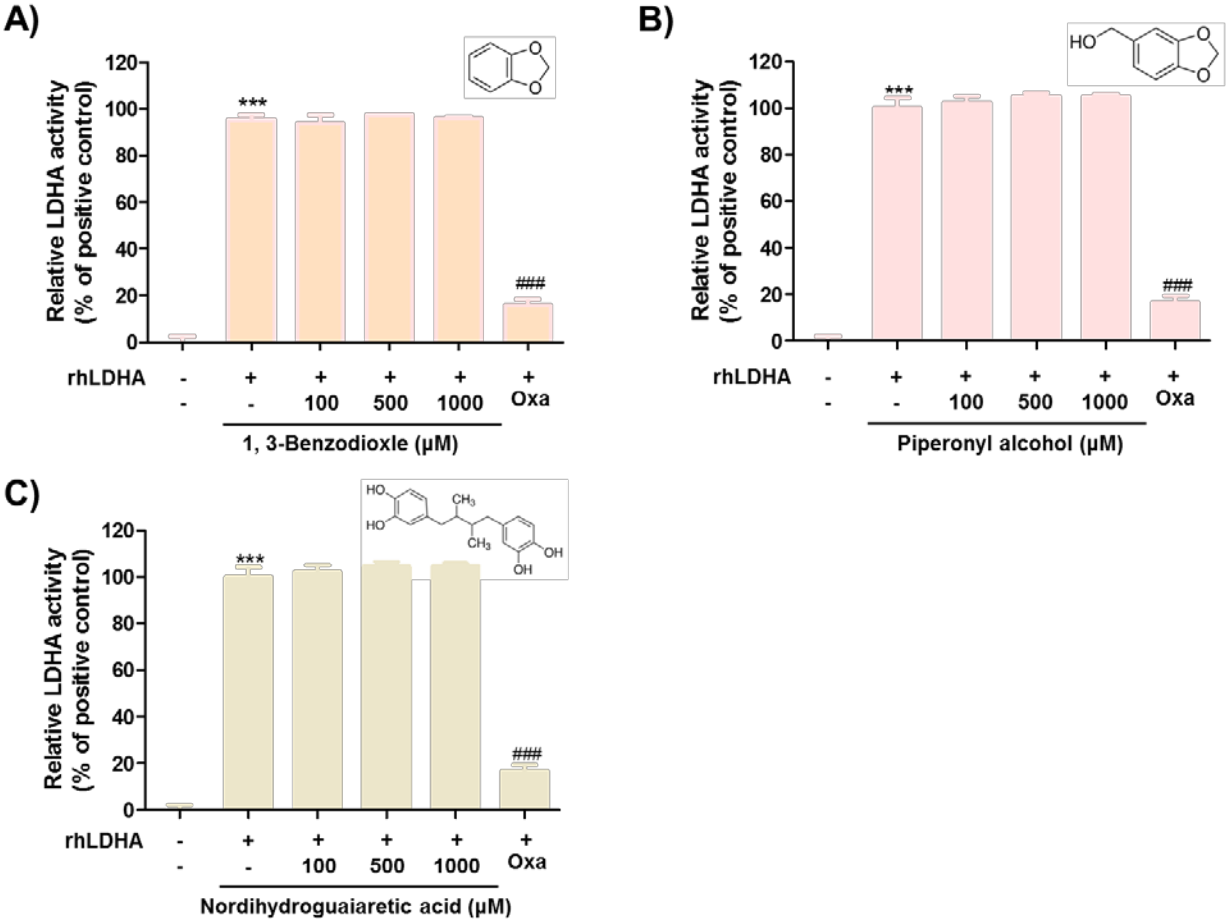

Figure S1. Inhibitory effect on LDHA activity**.** The inhibition of LDHA activity was analyzed using purified recombinant LDHA in presence of the indicated concentrations of (**A**) 1,3-benzodioxole, (**B**) piperonyl alcohol, or (**C**) nordihydroguaiaretic acid. Oxamate (50 mM) was used as positive control for LDHA inhibition. Results of three independent replicates are presented as means ± SD. Statistic tests were performed using a one-way analysis of variance (ANOVA). *** $p < 0.001$ compared to the negative control (first column). *** $p < 0.001$ and ### $p < 0.001$ compared to the positive control (second column). LDHA: lactate dehydrogenase A; SD: standard deviation.

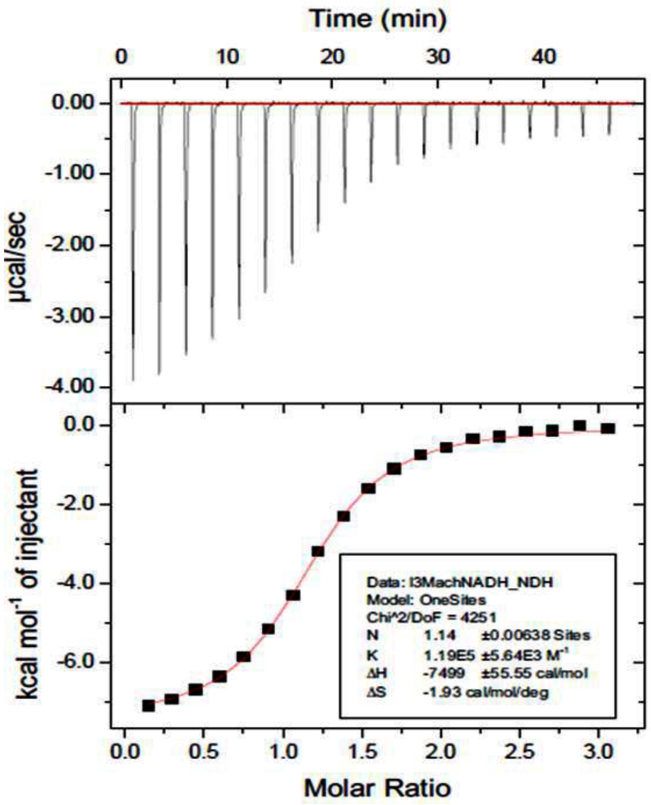

Figure S2. Isothermal titration calorimetry (ITC) analysis of the LDHA and NADH. The NADH was titrated into the LDHA solution. LDHA: lactate dehydrogenase A; NADH: 1,4-dihydronicotinamide adenine dinucleotide.

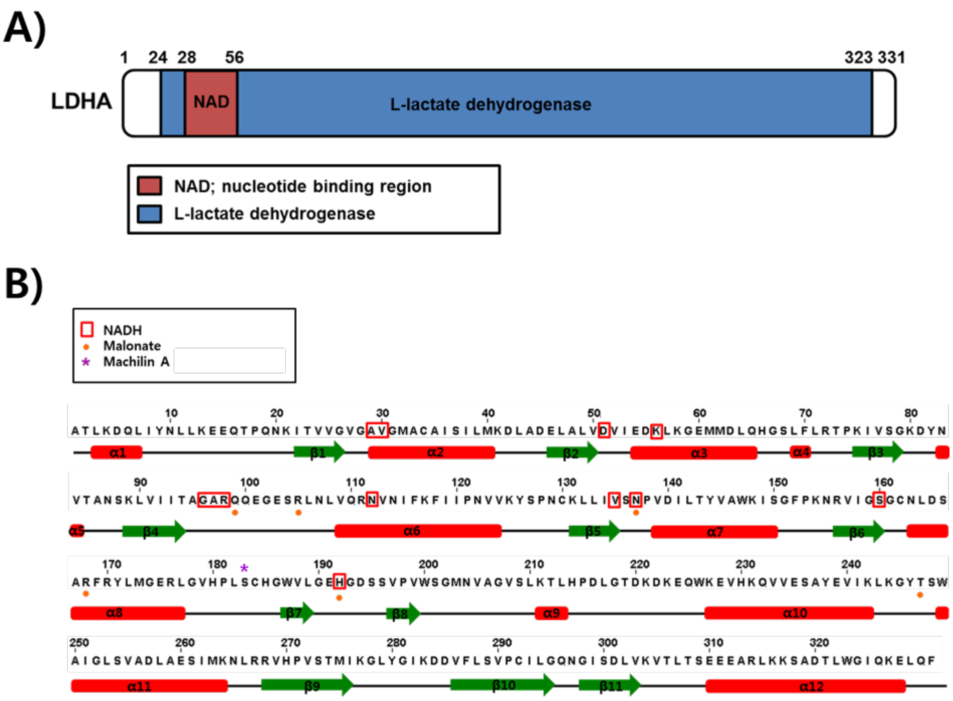

Figure S3. *Cont.*

C)

Ligands	Malonate	NADH	Machilin A
Structure	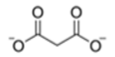	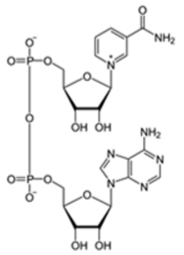	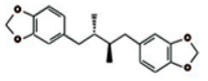
MW(Da)	102.4	665.4	326.4

Figure S3. Structures of LDHA with malonate/NADH/Machilin A. (**A**) Schematic diagram showing the domain of LDHA (1-331) is shown. (**B**) The secondary structure of LDHA is shown. Secondary elements are shown by arrows (β-sheets) and coils (α-helix). The loops are shown as black lines. Binding residues with malonate/NADH/Machilin A are indicated by red points, boxes, and stars, respectively. Structures of LDHA with malonate/NADH/Machilin A. (**C**) The chemical structures of malonate, NADH, and machilin A are shown. LDHA: lactate dehydrogenase A; NADH: 1,4-dihydronicotinamide adenine dinucleotide.

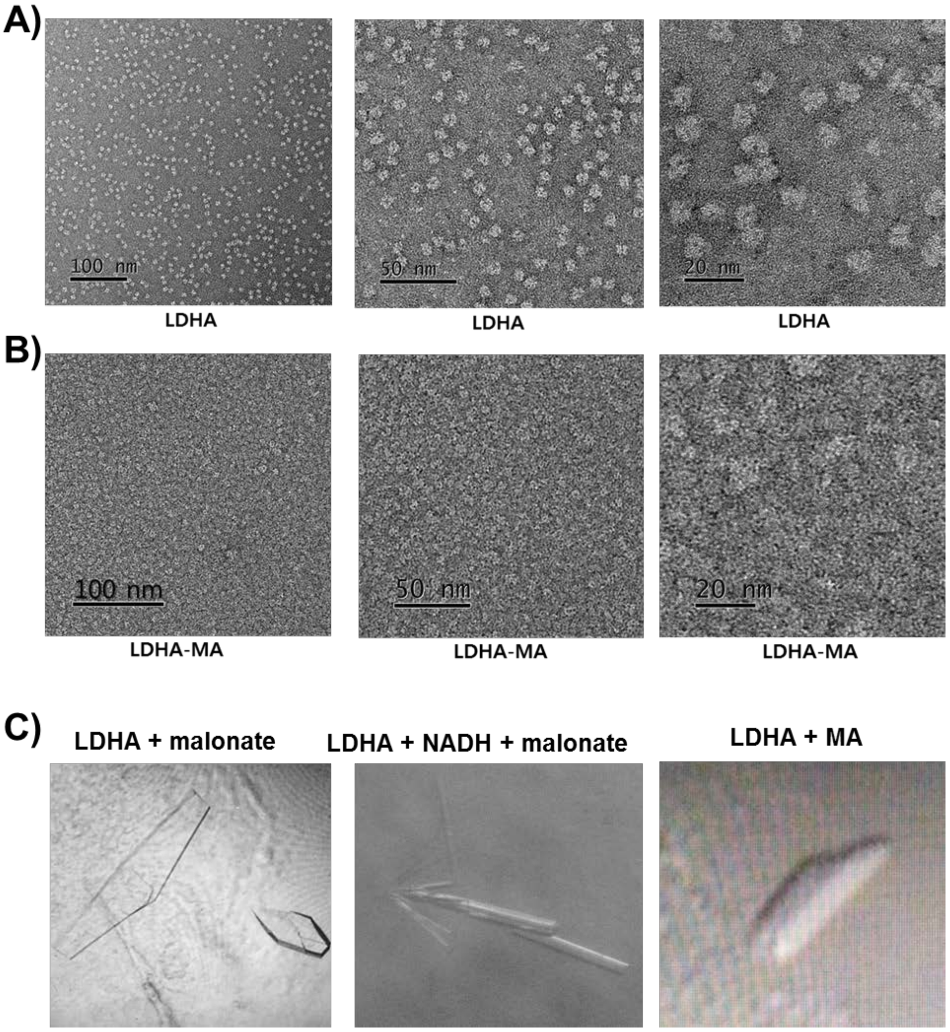

Figure S4. Visualization of LDHA tetramer-like shape by electron microscopy and crystal shapes of LDHA complexes. Typical electron micrograph area of LDHA negatively stained with uranyl acetate.

(**A**) LDHA with malonate. (**B**) LDHA with MA. (**C**) Crystals of LDHA with malonate/NADH/Machilin A complexes are shown. Scale bar. 100μm. LDHA: lactate dehydrogenase A; NADH: 1,4-dihydronicotinamide adenine dinucleotide.

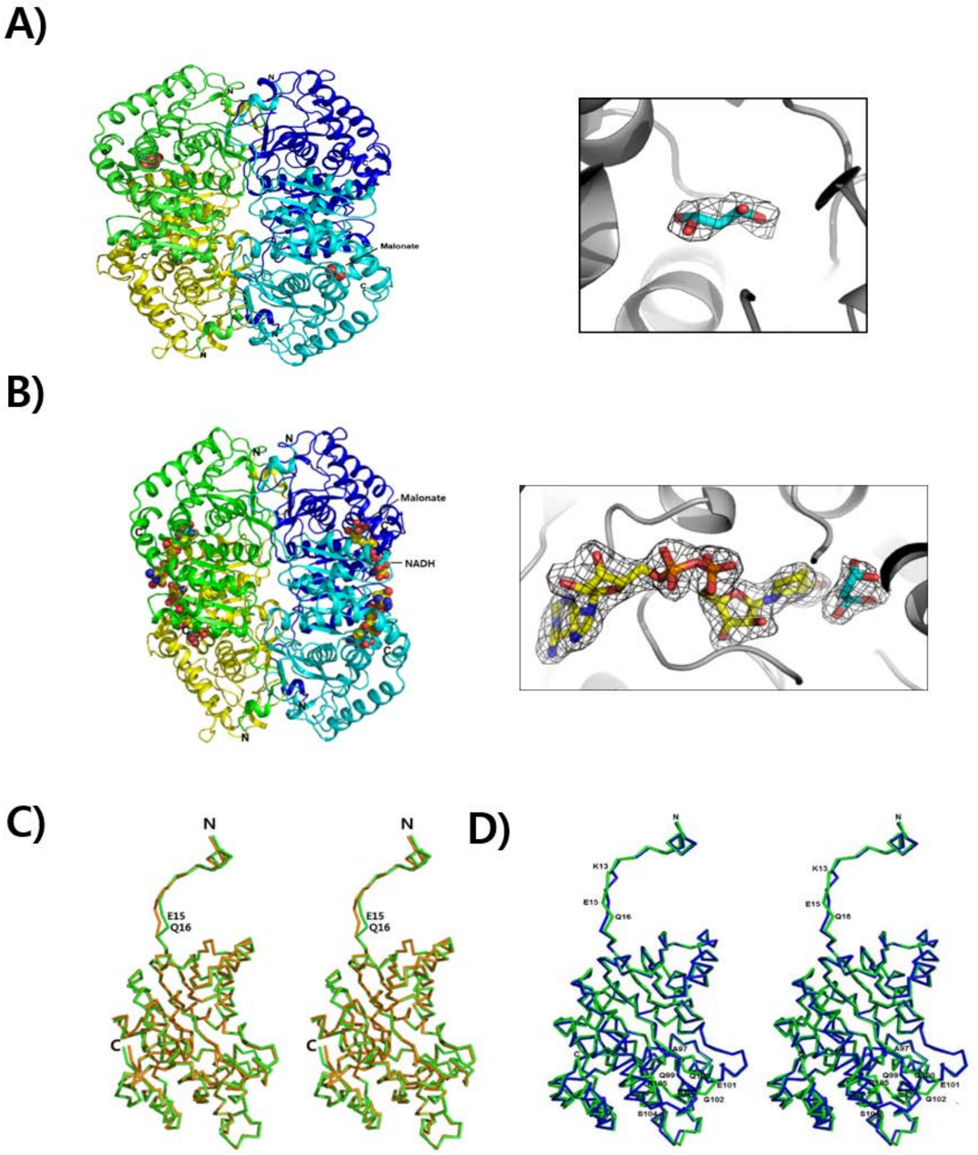

Figure S5. Ribbon diagrams, superpositions, and electron density maps of LDHA structures. (**A,B**) Tetrameric structures of LDHA with malonate/NADH are shown. Chains A, B, C and D are colored green, yellow, blue, and cyan, respectively. The LDHA molecules are color coded with C in green, O in red, and N in blue. The 2F_o-F_c electron density maps of LDHA with malonate and NADH are contoured at 1σ (gray) (**C**) Superposition of the LDHA complexes with malonate and NADH is shown. (**D**) Superposition of LDHA complexes with machilin A and NADH is shown. LDHA: lactate dehydrogenase A; NADH: 1,4-dihydronicotinamide adenine dinucleotide.

.

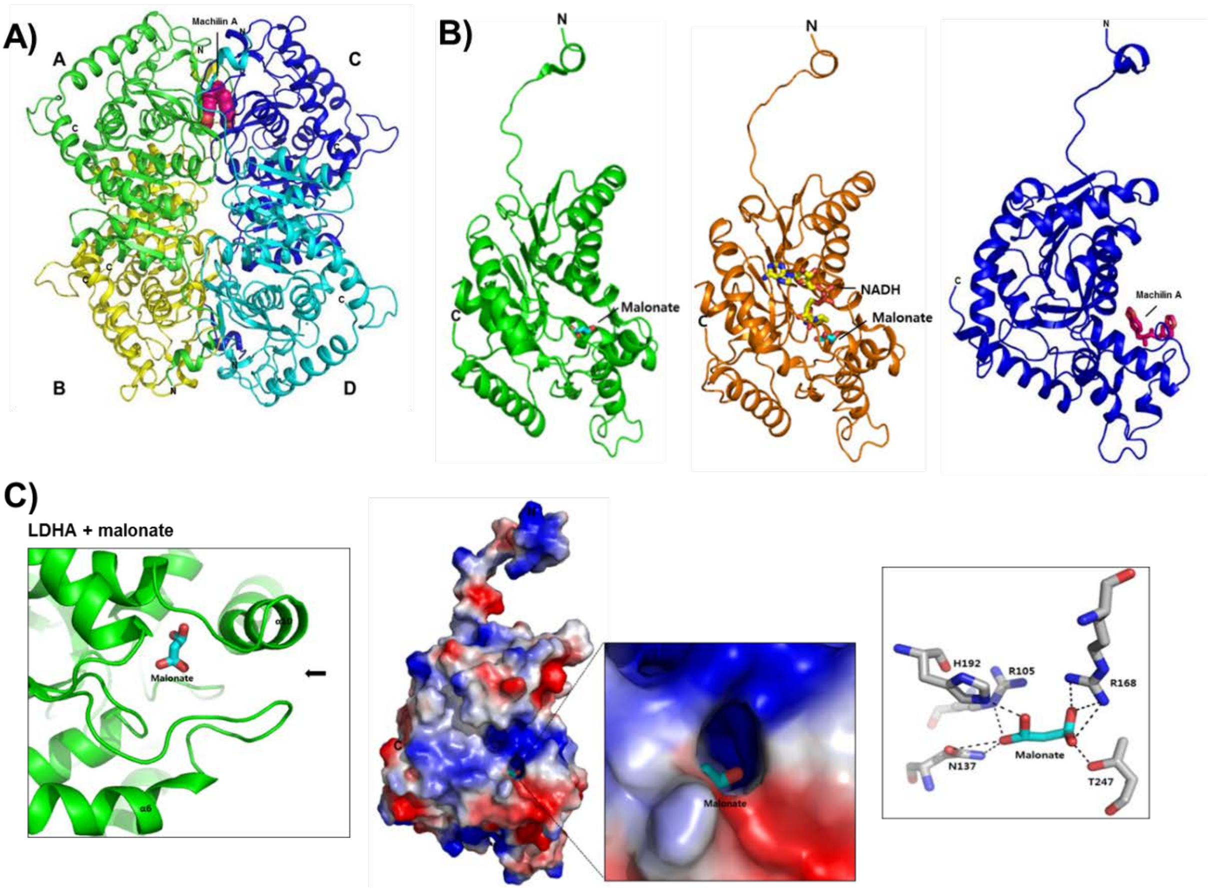

Figure S6. Structures of human LDHA with malonate and LDHA/Machilin A. (**A**) Tetrameric structure of human LDHA/Machilin A. Chains A, B, C, and D of LDHA are shown in green, yellow, blue, and cyan, respectively. (**B**) The C-chains of LDHA with malonate, NADH, and machilin A are shown, respectively. (**C**) The active-site loop region of LDHA-malonate is shown as closed form for substrate binding (left panel). Shown are the complexes of LDHA and malonate (middle panel) The relative distribution of the surface charge is shown with the acidic region in red, the basic region in blue, and the neutral region in white. The side chains of several amino acids of LDHA with malonate is shown (right panel). Hydrogen bonds in LDHA complexes with malonate is shown as black dotted lines. LDHA: lactate dehydrogenase A; NADH: 1,4-dihydronicotinamide adenine dinucleotide.

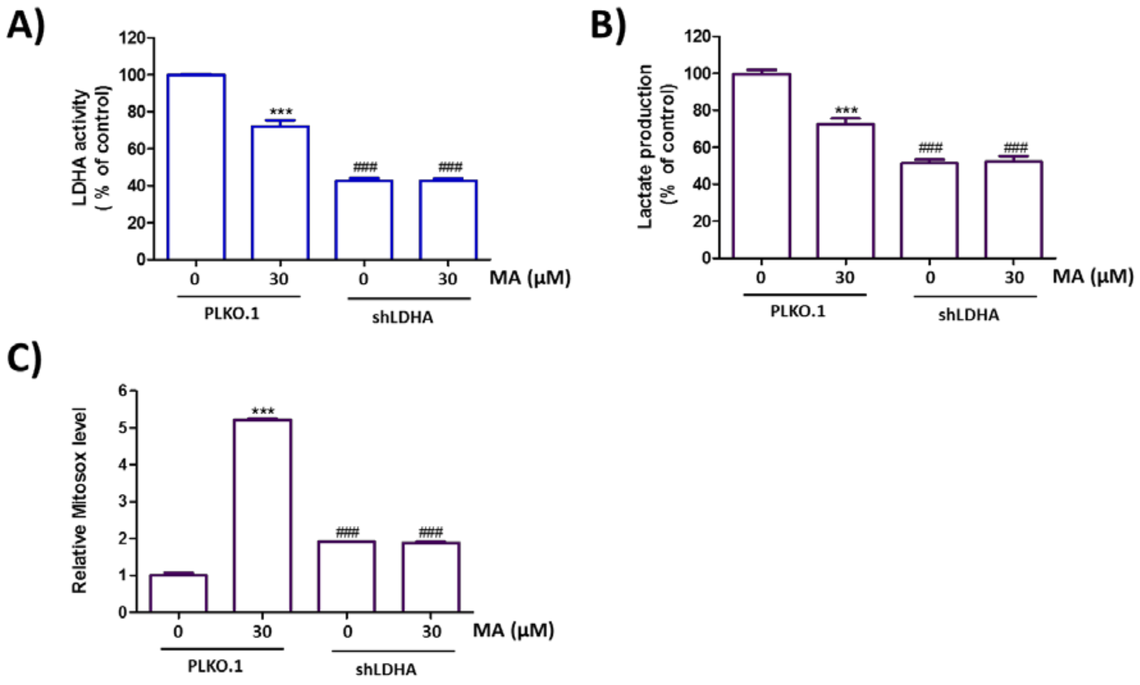

Figure S7. LDHA is crucial for the MA effect on suppression of cancer cells. HT29 cells transfected with PLKO.1 or shLDHA were treated with Machilin A (30 μM) for 48 h. (**A**) LDHA activity was examined using cell lysates. (**B**) Lactate production was investigated from culture media of HT29 cells. (**C**) Mitosox was labeled with red fluorescence and analyzed by FACS. Data are presented as mean ± SD. Statistic tests were performed using a one-way ANOVA. *** $p < 0.001$ compared to the control transfected with PLKO.1 (first column). ### $p < 0.001$ compared to the sample transfected with PLKO.1 and treated with MA (second column). LDHA: lactate dehydrogenase A; FACS: Fluorescence-activated cell sorting; ANOVA: Analysis of variance; SD: standard deviation.

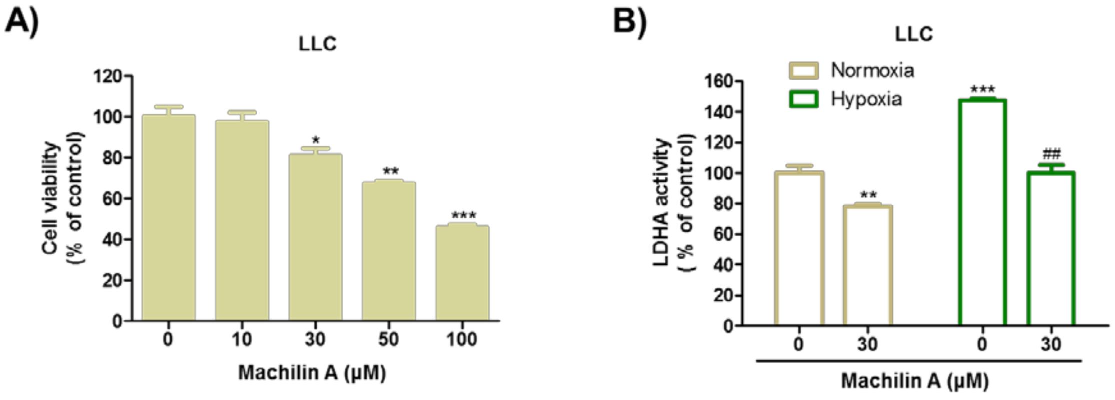

Figure S8. The cell viability and LDHA activity of machilin A on LLC cells. (**A**) LLC cells were treated with machilin A (30 μM) for 48 h under normoxic and hypoxic conditions. The activity of LDH was investigated using cell lysates. Data are presented as mean ± SD. Statistic testing was performed using a Student's t-test. *** $p < 0.001$ compared to the control under normoxia (first column). ## $p < 0.01$ compared to the control under hypoxia (third column). (**B**) LLC cells were treated with the indicated concentrations of machilin A for 48 h. Cell viability was evaluated using an MTT assay. Results of three independent replicates are shown as means ± SD. Statistic testing was performed using a one-way ANOVA. * $p < 0.05$, ** $p < 0.01$, and *** $p < 0.001$, compared to the control. LDHA: lactate dehydrogenase A; SD: standard deviation.

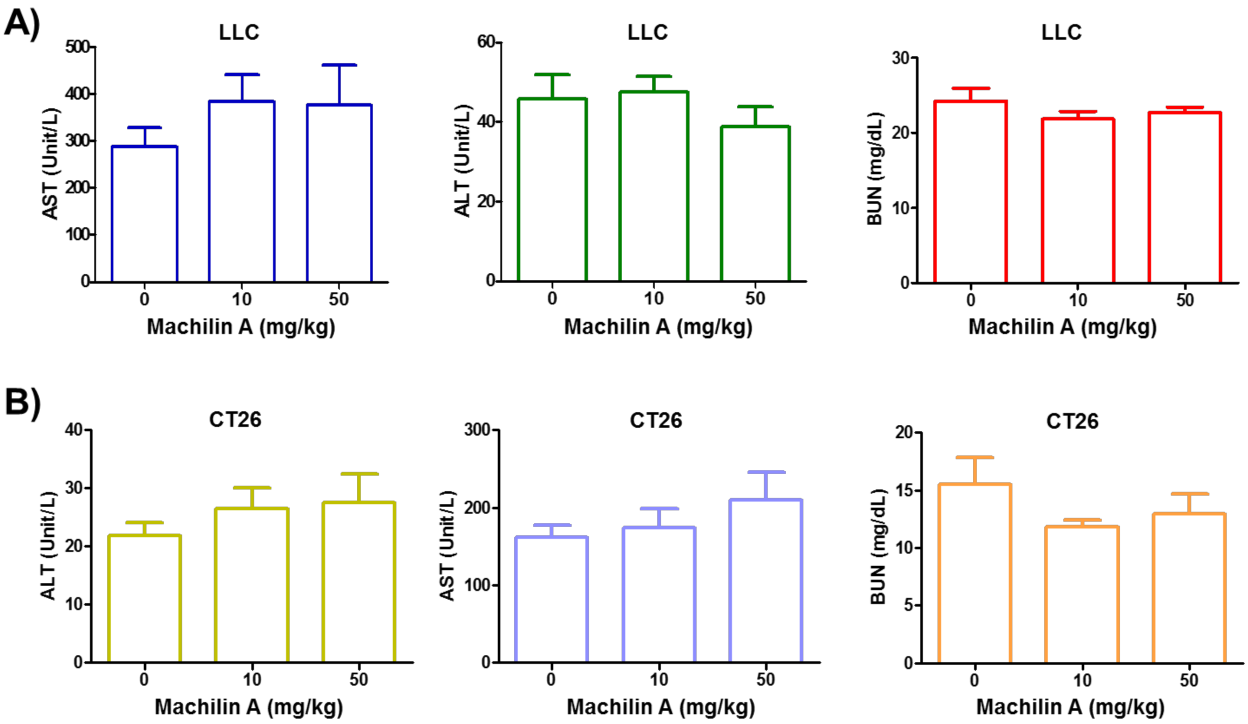

Figure S9. The effects of machilin A on liver or kidney functioning. (**A**) LLC and (**B**) CT26 cells were inoculated subcutaneously into mice. After inoculation, the indicated dosages of machilin A were intraperitoneally injected into mice daily. Thirteen days after inoculation of tumor. The blood of each mouse was collected from the retro-orbital plexus, and then the sera from blood were prepared. To investigate the toxicity of machilin A on liver and kidney of mice, biochemical analysis on the aspartate aminotransferase (AST), alanine aminotransferase (ALT), creatinine, and blood urea nitrogen (BUN) were performed by commercial service by Green Cross Co.

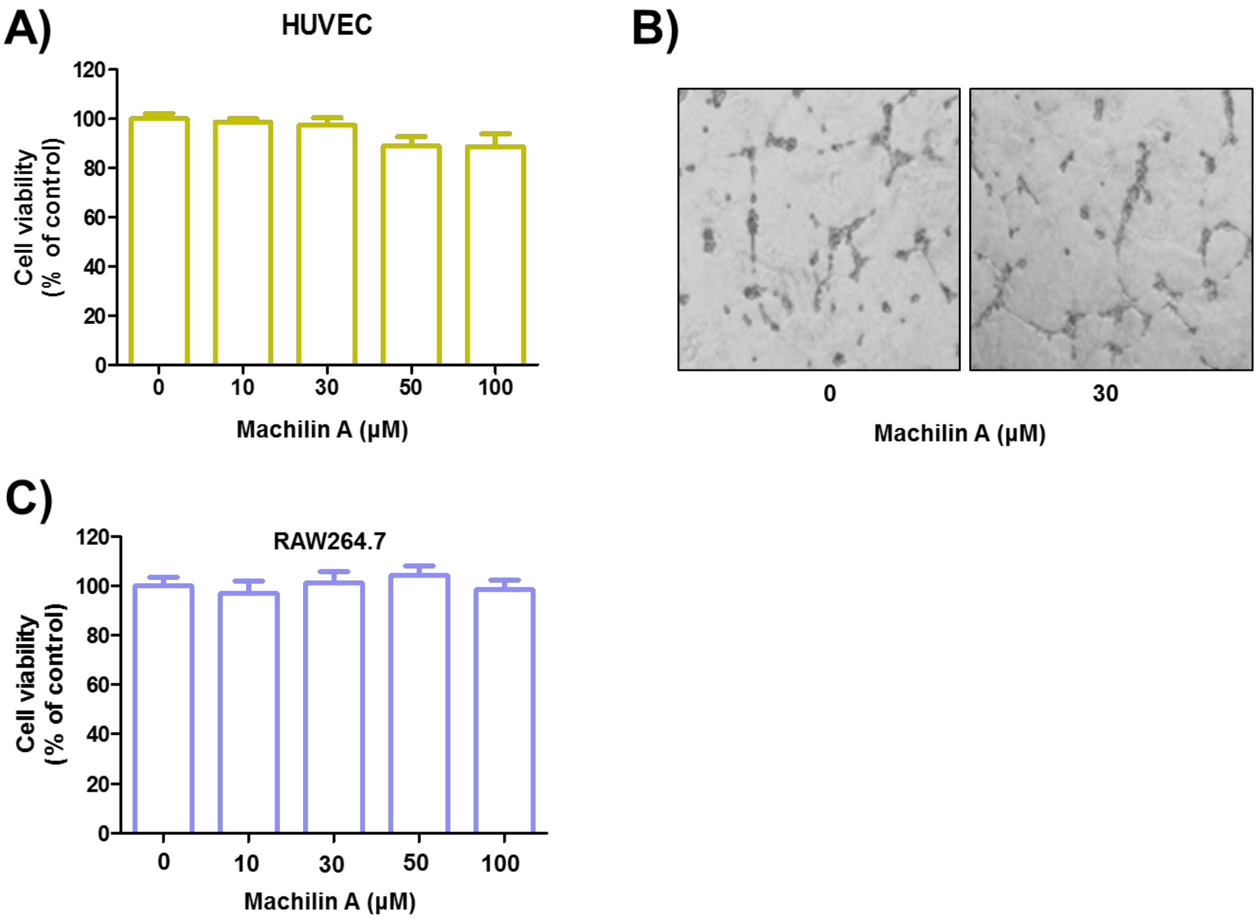

Figure S10. The viabilities of HUVECs and RAW264.7 cells on MA. (**A**) HUVECs were treated with indicated concentration of machilin A for 24 h. The viabilities of cells were evaluated by MTT assay. (**B**) HUVECs seeded to the Matrigel-coated wells treated with machilin A for 24 h. (**C**) Raw 264.7 macrophage cells were treated with indicated concentration of machilin A for 24 h. The viabilities of cells were evaluated by MTT assay.

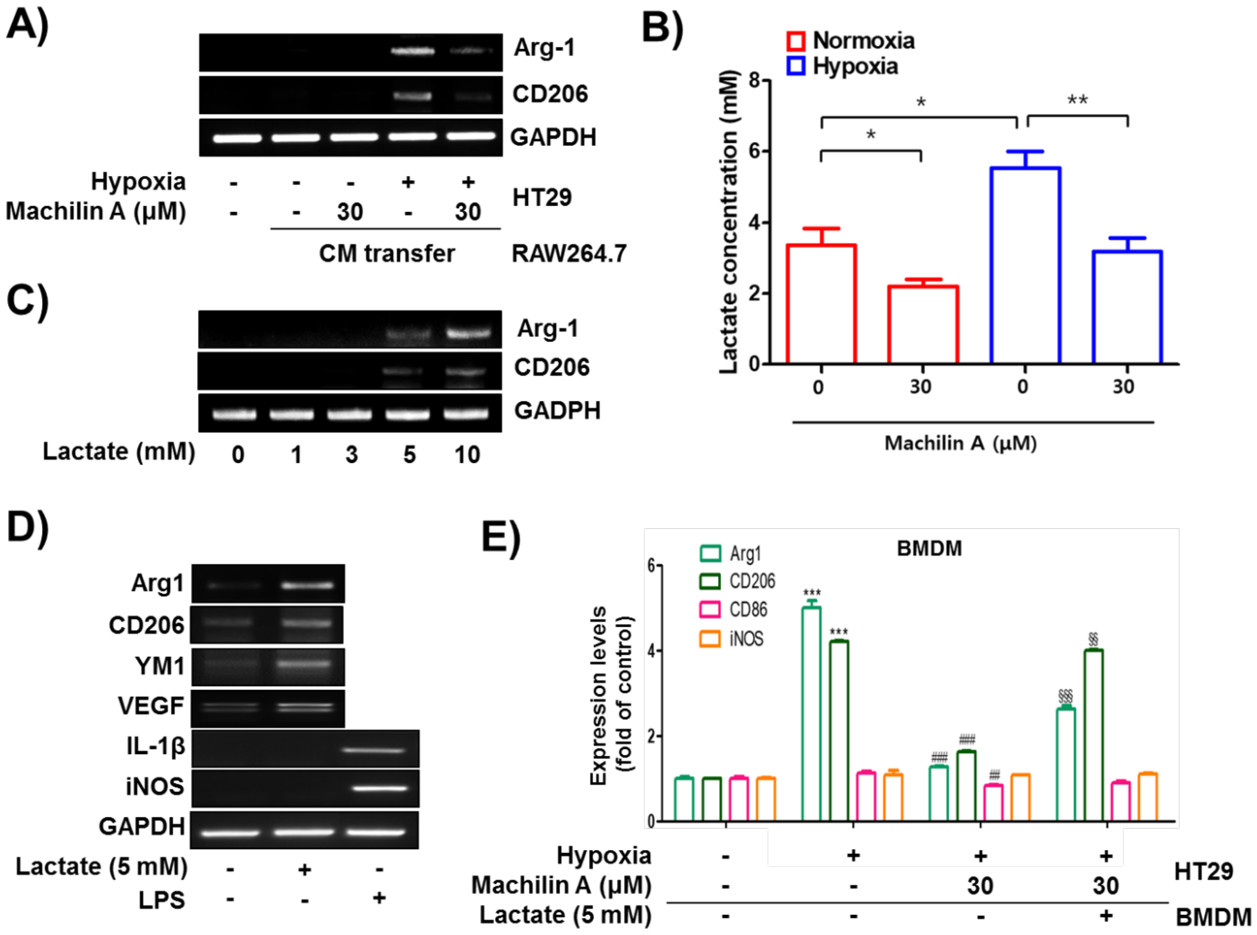

Figure S11. Suppression of tumor-derived lactic acid in MA-treated cancer cells. (**A**) The HT29 cancer cells were treated with or without machilin A (30 μM), and incubated in normoxic or hypoxic condition. At 48 h after incubation, Raw 264.7 macrophage cells was treated with the conditioned medium (1 mL) harvested from cancer cells for 24 h. The expressions of *Arg-1* and *CD206* mRNAs were determined by RT-PCR. *GAPDH* mRNA expression was used for internal control. (**B**) To determine effect of machilin A on lactate production in cancer cells using NMR spectroscopy, HT29 colorectal cancer cells (5×10^5 cells/2mL) were treated in presence of machilin A (30 μM) in normoxia and hypoxia conditions. Production of lactate was measured by NMR spectroscopy. The results from 3 independent experiments are shown as the means ± SD. * $p < 0.05$ compared to the normoxia control (1st column). ** $p < 0.01$ compared to the hypoxia control (3rd column). (**C**) Raw 264.7 macrophage cells were treated with indicated concentration of lactic acid for 24 h. The expressions of *Arg-1* and *CD206* mRNAs were determined by RT-PCR. *GAPDH* mRNA expression was used for internal control. (**D**) Raw 264.7 macrophage cells were treated with lactic acid or LPS for 24 h. The expressions of *Arg-1*, *CD206*, *YM1*, and *VEGF* mRNAs were determined by RT-PCR. *GAPDH* mRNA expression was used for internal control. (**E**) The HT29 cancer cells were treated with or without machilin A (30 μM), and incubated in hypoxic condition. At 48 h after incubation, the conditioned medium (1 mL) harvested from cancer cells was added to bone marrow-derived macrophage in the presence or absence of lactate (5 mM). The expressions of ARG-1 and CD206 proteins as M2 markers and the expressions of CD86 and iNOS proteins as M1 markers were determined by FACS. Data are presented as mean ± SD. Data were also statistically compared using the Student's t-test. *** $p < 0.001$ compared to No.1. ## $p < 0.01$ and ### $p < 0.001$ compared to No.2. §§ $p < 0.01$ and §§§ $p < 0.001$ compared to No.3.

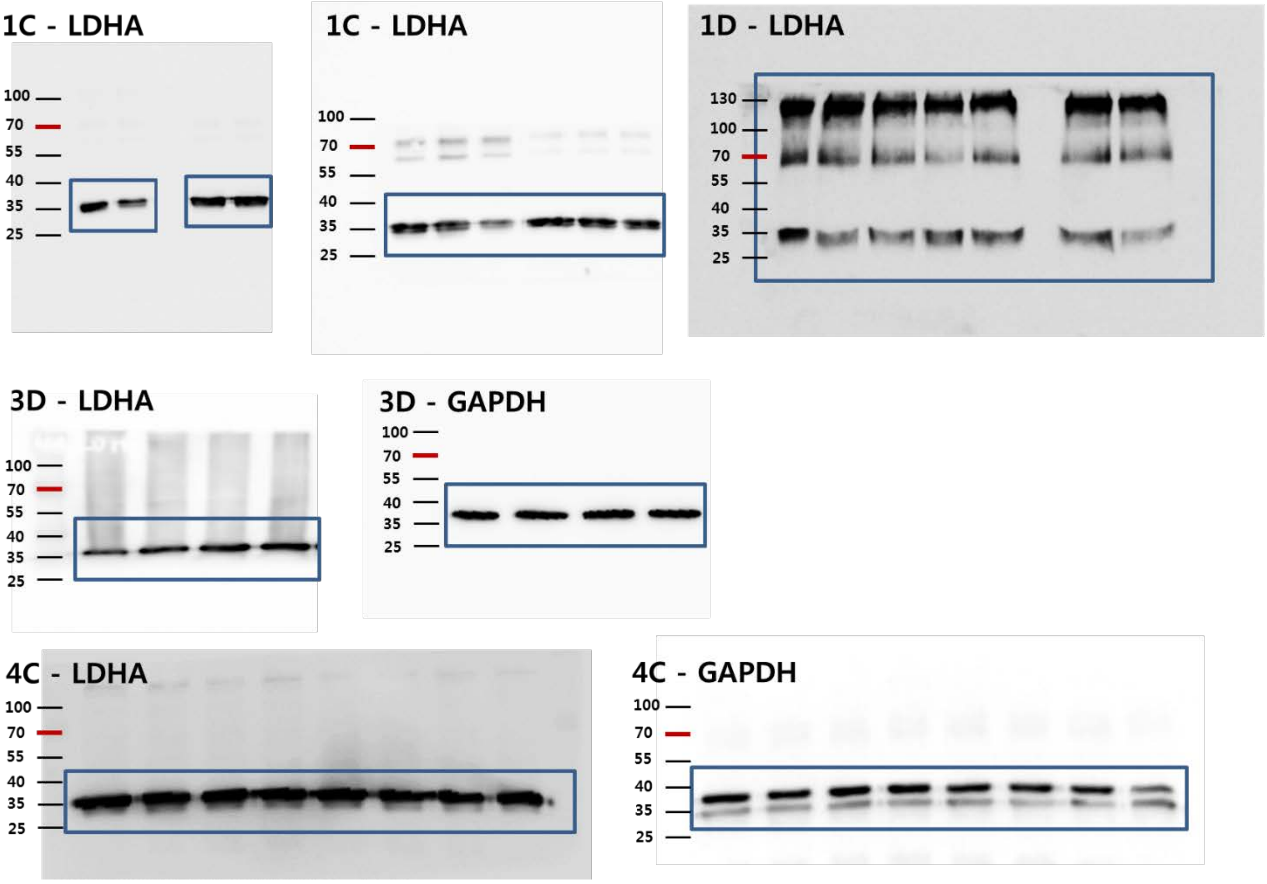

Figure S12. Uncropped immunoblots of the most important blots.

Table S1. Crystallographic Statistics.

Data Collection Statistics	(PDB ID: 5ZJE)	(PDB ID: 5ZJD)	(PDB ID: 5ZJF)
Crystal	LDHA-malonate	LDHA-NADH-malonate	LDHA-Machilin A-malonate
Space group	$P2_1$	$P2_1$	$P2_1$
Unit cell dimensions (Å)	a = 85.6, b = 219.3, c = 112.8 α = 90° β = 92.3° γ = 90°	a = 64.7, b = 265.1, c = 87.2 α = 90° β = 102.9° γ = 90°	a = 134.2, b = 141.5, c = 144.1 α = 90.0° β = 110.2° γ = 90.0°
Resolution (Å)	30.2–2.93 (2.96–2.93)	30-2.39 (2.42-2.39)	37.1–2.6 (2.63–2.60)
Completeness (%)	97.5 (100.0)	99.9 (100.0)	99.4 (95.2)
Observed reflections	1694609	1212123	560845
Unique reflections	86824	103853	153798
I/σ (I)	11.4 (1.36)	18.3 (1.34)	19.0 (1.35)
R_{merge} (%)[a]	16.8 (38.5)	9.4 (30.9)	7.1 (21.2)
Redundancy	3.7	3.8	1.0
**Refinement statistics**			
Resolution range (Å)	30–2.93	29.8–2.39	37–2.6
R_{cryst}/R_{free} (%)[b]	18.8/28.4	19.1/25.3	18.1/23.9
Proteins/ Water	3972/346	2648/415	3975/371
NADH (mols)	0	8	0
Machilin A (mols)	0	0	1
Malonate (mols)	6	8	0
Rmsd bond length (Å)/angles (°)	0.010/1.241	0.008/1.303	0.008/1.102
Average B-factor (Å^2)	51.5	39.5	51.9
**Ramachandran plot (%)**			
Most favored region	87.8	93.0	94.9
Additional allowed region	9.02	5.74	3.90
Generously allowed region	3.14	1.29	1.17

Values in parentheses are for the highest resolution shell. [a]$R_{merge} = \sum|I_i I_m|/\sum I_i$, where I_i is the intensity of the measured reflection and I_m is the mean value of all symmetry-related reflections. [b]$R_{cryst} = \Sigma||F_{obs}|-|F_{calc}||/\Sigma|F_{obs}|$, where F_{obs} and F_{calc} denotes the observed and calculated structure factor amplitude. $R_{free} = \sum_T||F_{obs}|-|F_{calc}||/\Sigma_T|F_{obs}|$, here T is a test data set of about 5% of the total reflections randomly chosen and set aside prior to refinement. LDHA: lactate dehydrogenase A; NADH: 1,4-dihydronicotinamide adenine dinucleotide.

Table S2. Interaction distances of LDHA with malonate/NADH/Machilin A.

	Interactions	Distance (Å)		Interactions	Distance (Å)
				N (Ala29)–O2A(NADH)	3.32
				N (Ala29)–O3	3.32
				N (Val30)–O1N	3.03
				OD1 (Asp51)–O2B	2.61
				OD2 (Asp51)–O2B	2.97
				OD2 (Asp51)–O3B	2.57
				NZ (Lys56)–O3B	2.65
				O (Gly96)–O5D	3.40
				O (Ala97)–O3	3.11
	NE (Arg105)–O2(Mal)	3.34		NH1 (Arg98)–O2N	3.15
	ND2 (Asn137)–O2	2.91		OD1 (Asn112)–O3	3.24
	OD1 (Asn137)–O2	3.41		O (Val135)–N7N	2.87
LDHA + malonate	NH1 (Arg168)–O1	3.03	LDHA + NADH + malonate	ND2 (Asn137)–O2D	3.10
	NH1 (Arg168)–O3	3.36		ND2 (Asn137)–N1N	3.28
	NH2 (Arg168)–O1	3.09		OG (Ser160)–N7N	3.35
	NE2 (His192)–O4	2.53		NE2 (His192)–O7N	2.95
	OG1 (Thr247)–O3	2.56		NE (Arg105)–O2(Mal)	2.96
				NE (Arg105)–O4	2.79
				NH2 (Arg105)–O4	2.72
				ND2 (Asn137)–O2	2.52
				NH1(Arg168)–O3	2.87
				NH1(Arg168)–O1	2.65
				NH2(Arg168)–O1	2.72
				NE2 (His192)–O2	3.05
				NE2 (His192)–O4	2.70
				OG1 (Thr247)–O3	2.84
LDHA + Machilin A	OG (Ser183)–O2	3.01			

LDHA: lactate dehydrogenase A; NADH: 1,4-dihydronicotinamide adenine dinucleotide.

Supplementary Methods

1.1. Electron Microscopy

Purified lactate dehydrogenase A (LDHA) (concentration, 0.5 mg/mL) was applied to a glow-discharged copper grid covered with a continuous carbon film. The sample negative stained with 1% (w/v) uranyl acetate for 1 min. Images were collected at a nominal magnification of 67,000x, 110,000x and 220,000x by a Tecnai G2 Spirit electron microscope.

1.2. Sample Preparation of NMR Spectroscopy

350 μL of media sample was transferred to micro centrifuge tube containing 350 μL of deuterated sodium phosphate buffer containing 4 mM of TSP-d_4(3-(trimethylsilyl) propionic-2,2,3,3-d4 acid sodium salt). 630 μL of cell lysate sample was transferred to micro centrifuge tube containing 70 μL of deuterated sodium phosphate buffer containing 20 mM of TSP-d_4. Each sample was transferred to 5-mm nuclear magnetic resonance (NMR) tube. Trimethylsilylpropanoic acid (TSP) was used as a quantitative reference (2 mM) and standard of chemical shift (0.00 ppm). Each sample was transferred to 5-mm NMR tube.

1.3. NMR Spectroscopy

All spectra were acquired at 600.167 MHz Agilent NMR spectrometer. CPMG (Carr-Purcell-Meiboom-Gill) pulse sequence was used because of suppression of water and high molecular mass

compounds. The acquisition time was 1.704 sec, relaxation delay was 1.5 sec and 128 transient were collected. Lactate was quantified using Chenomx NMR Suite 7.1 professional with the Chenomx 600 MHz library database (Chenomx Inc., Edmonton, Alberta, Canada,).

1.4. Isothermal Titration Calorimetry

Isothermal titration calorimetry (ITC) measurements were carried out using MicroCal auto_iTC200 (GE healthcare, Chicago, IL, USA) at 25 °C. The purified LDHA protein was dialyzed in buffer containing 50 mM Tris-HCl (pH 8.0) and 200 mM NaCl at a concentration of 0.1 mM. NADH was solubilized in the same buffer at concentrations of 1 to 2 mM. The NADH was titrated into the LDHA solution onto an ITC200 microcalorimeter cell. The raw data were integrated and normalized using MicroCal Origin 7.0 software (GE healthcare). The determined binding affinity (KA) and enthalpy changes (ΔH) values were used to calculate ΔS from the standard thermodynamic equation.

1.5. Flow Cytometry

For generation of bone marrow-derived macrophage (BMDM), bone marrow cells were extracted from C57BL/6 mice by surgery, and treated with M-CSF (10ng/ml) for 7days. BMDM cells were incubated with the indicated concentration of culture media with MA and lactate or not. The cells were washed with phosphate buffered saline (PBS) and fixed in 3.7% formaldehyde in PBS for 1h. After washing two times with PBS, the samples were incubated with primary antibody against CD206 (ab64693, Abcam, Cambridge, UK), Arg-1 (#9819, Cell Signaling, Danvers, MA, USA), CD86 (sc-28347, SantaCruz, Clara, CA, USA), or iNOS (#482728, Calbiochem, St. Louis, MO, USA) in 5% bovine serum albumin (BSA; Sigma-Aldrich, St. Louis, MO, USA) for 2 h. The cells were washed again and incubated with secondary antibody for 1 h. The samples were examined on a BD FACSCanto II flow cytometer (BD Biosciences, San Jose, CA, USA).
